# Connectedness to Family, School, and Neighborhood and Adolescents’ Internalizing Symptoms

**DOI:** 10.3390/ijerph182312602

**Published:** 2021-11-29

**Authors:** Danielle R. Eugene

**Affiliations:** School of Social Work, University of Texas at Arlington, Arlington, TX 76019, USA; danielle.eugene@uta.edu

**Keywords:** adolescent mental health, internalizing symptoms, protective factors, social connectedness, social environment

## Abstract

In the U.S., there is a strong national interest in social connectedness as a key determinant in promoting positive well-being in adolescents through building strong bonds and creating protective relationships that support adolescent mental health. To this end, this study examined whether, and to what extent, specific types of connectedness to family, school, and neighborhood were associated with internalizing symptoms (i.e., depression and anxiety) among a diverse sample of adolescents from disadvantaged backgrounds. The sample (*n* = 2590) was majority male (51%), with an average age of 15.6 years, and identified as Black (49%) and Hispanic/Latino (26%). The results revealed that adolescents who reported strong connections to their parent (*β* = −0.128, *p* < 0.001), school (*β* = −0.222, *p* < 0.001), and neighborhood (*β* = −0.116, *p* = 0.003) were more likely to report lower levels of depressive symptomology, with school connectedness exerting a greater influence. In addition, parent connectedness (*β* = −0.157, *p* < 0.001) and school connectedness (*β* = −0.166, *p* < 0.001) were significantly related to teen anxiety; however, neighborhood connectedness was not (*β* = −0.123, *p* = 0.087). The findings have important implications, which are discussed.

## 1. Introduction

According to the Centers for Disease Control and Prevention (CDC), the number of U.S. adolescents reporting internalizing symptomology continues to rise [1]. In 2019, the most recent year in which data was available, more than one-third of youth reported persistent feelings of depression which was a 40% increase since 2009. Also, one in six youth reported having suicidal ideations compared to one in eight youth in 2009 [2]. In another U.S. study, [3] found that more than 8.4% of youth aged 6 to 17 had a clinical diagnosis of depression or anxiety. Depression and anxiety are internalizing mental disorders with their onset peaking during early adolescence [4,5]. Depression is characterized as persistent sadness, hopelessness, or an irritable mood and anxiety is often referred to as excessive fear or worry [5]. These conditions have been associated with serious negative outcomes for youth including lower educational achievement, illicit substance use, risky sexual behavior, delinquent behavior, and increased suicide risk [6]. Beyond this, these internalizing symptoms frequently persist into adulthood and are associated with an increased risk of co-occurring disorders, criminal involvement, lower wage earnings, and early mortality [7]. 

Certain populations of youth are at higher risk of mental disorders due to greater exposure and vulnerability to unfavorable social, economic, and environmental circumstances. Considerable and growing evidence show that mental health symptoms in adolescence and many common mental disorders are associated with family conditions (e.g., socioeconomic status, education, employment, or material disadvantage), parenting behaviors/attitudes, social isolation, and environmental factors such as neighborhood deprivation or safety issues [8]. An important systematic review of the literature revealed that the prevalence of depressed mood or anxiety was 2.5 times higher among youth aged 10 to 15 years with low socioeconomic status than among youth of the same age with high socioeconomic status [9]. In addition, epidemiological studies on the distribution of positive mental health revealed that poor mental health was found in poorer groups and among those who reported weak social support [10]. Taking action to improve the conditions of daily life during adolescence is necessary and can be enhanced by the social and emotional support and positive interactions with family, school, and the larger community [8,11].

There is an emerging interest in social connectedness as a key determinant in promoting positive well-being in adolescents through building strong bonds and creating protective relationships that support adolescent mental health [1,11,12]. Connectedness has commonly been referred to as a sense of belonging or a psychological bond that a person may feel towards other people or groups within social contexts such as family, school, and communities [13,14]. Conceptualized within the ecological framework [15], social contexts, classified from most proximal to most distal, interact to influence various aspects of adolescent mental well-being. This includes the microsystems (the most proximal environmental context such as family and peers); mesosystems (the linkages between microsystems); exosystems (broader systems with which adolescents have contact (including schools and communities); and macrosystems (such as distal political, economic, or cultural systems) [15,16]. An increasing number of studies have documented significant associations between connectedness in a variety of social contexts and adolescent mental health [11,12,17,18,19]. However, lacking are studies that simultaneously assess major domains of connectedness (e.g., family, school, and neighborhood) in relation to internalizing symptomology during adolescence. This study was designed to address this shortcoming in the literature by examining whether, and to what extent, specific types of connectedness (to family, school, and neighborhood) were associated with internalizing problems (i.e., depression including suicidal ideation, and anxiety) among adolescents. Connectedness has the potential to be a target of interventions that are designed to increase protective factors for youth [13,20]. 

## 2. Literature Review

### 2.1. Connectedness as a Theoretical Basis

A sense of belonging is a fundamental and pervasive human need that is based on strong biological and psychological mechanisms [21]. Extensive evidence emphasizes the importance of feeling connected to others and social institutions for adolescent mental health and well-being [12,18,22]. The centrality of connectedness is embedded in various theories and models (e.g., attachment theory [23], family warmth and cohesion [24]) that support healthy connections as a fundamental need between youth and their families. Furthermore, research on social support, integration, and connection highlights the advantages of forming positive relationships outside of the immediate family [11,12,25]. As a growing framework for intervention, connectedness is a malleable mechanism that contributes to an improved sense of belonging, increase in social networking, and potential engagement in one’s social environment [22]. Not only is social connectedness instrumental in optimal outcomes for youth, but a lack of such connections have been linked to poor mental and physical health among youth [18]. As such, enhancing adolescent’s connections within their social environment may reduce potential risk factors while promoting positive mental health outcomes. 

### 2.2. Connectedness and Internalizing Problems

#### 2.2.1. Parent Connectedness

Research has demonstrated the influential effect of familial relationships on youth mental health outcomes [26]. In particular, research suggests that vulnerable youth who feel close to their parents have lower risk for internalizing disorders. For example, [25] found that youth perceptions of parent connection was inversely related with internalizing symptomology (e.g., anxiety/depression, withdrawal, and somatic complaints) among war-affected adolescents between the ages of 11–17 years. Similarly, [18] found that high-risk youth (aged 12 to 15) who reported stronger connections to their parents were more likely to report lower levels of depression and suicidal behaviors. Empirical findings suggest the protective role of parent-child connectedness in vulnerable youth populations. 

#### 2.2.2. School Connectedness

Connectedness to schools has been an overlooked potential protective factor for adolescent mental health and well-being [27]. It has, however, often been largely associated with achievement outcomes for students [28,29,30]. Emerging research has demonstrated the link between students’ connections to school and their mental health functioning. For example, research has shown that U.S. adolescents with an average age of 15.5 years who reported an increased sense of school connection reported less depressive symptoms including suicidal ideations and less anxiety [17]. Another study that was conducted with Danish high schoolers found that social disconnectedness at school was positively associated with depressive and anxiety symptoms, suicidal ideation, sleep disturbances, and stress, and was negatively associated with mental well-being [31]. Recent empirical findings point to the benefits of youth connections in the school environment in promoting positive mental health during adolescence. 

#### 2.2.3. Neighborhood Connectedness

Few studies have focused on the relationship between environmental factors, such as perceptions of neighborhood connectedness, and adolescent mental health. The existing literature shows that disordered and detached communities have been associated with increased rates of adverse mental health outcomes in adolescents [32]. Studies that have focused exclusively on the effects of neighborhood connectedness on internalizing symptomology present inconclusive findings. For example, [18] found that youth who reported increased connections to their neighborhood were more likely to report reductions in anxiety symptoms. However, in previous studies with adolescent samples, researchers indicated that neighborhood connectedness exhibited little to no effect on adolescent’s sense of psychological well-being [12] and internalizing problems [25]. In sum, more research is needed to contribute to the current literature and to understand the direct effects of connectedness to neighborhood on internalizing symptoms among adolescents. 

## 3. Current Study

Converging evidence suggests that connectedness to social contexts may act as a protective factor in reducing depressive and anxiety symptomology during adolescence. However, a significant gap in the literature points to a lack of empirical studies that simultaneously assess major domains of connectedness in relation to internalizing symptoms. The lacking of such investigation prevents a comprehensive assessment of perceptions of connectedness across social contexts and their influence on adolescent mental health. To this end, this study examined if specific types of connectedness to family, school, and neighborhood were associated with internalizing symptoms (i.e., depression and anxiety) among an adolescent sample that is disproportionately from minority and disadvantaged backgrounds. It is hypothesized that stronger connections to family and school would be associated with lower levels of depression and anxiety symptoms. Analyses examining neighborhood connectedness were exploratory in nature given the inconsistency in previous research regarding neighborhood influences. 

## 4. Methods

### 4.1. Data Source and Sample

The data were retrieved from the Fragile Families and Child Wellbeing Study (FFCWS) which includes a large sample of children that were born to unmarried parents, who are disproportionately from minority and disadvantaged backgrounds. This longitudinal birth cohort study used a stratified sampling method to recruit participants of parent-child dyads in 20 U.S. cities at the turn of the 21st century. Data collection occurred at a child’s birth, and subsequent waves were collected at ages 1, 3, 5, 9, and 15. For a more detailed description of FFCWS design and methods, refer to [33]. For the current cross-sectional study, the analytic sample (*n* = 2590) was drawn from age 15 interviews (data collected 2014–2017).

### 4.2. Measures

#### 4.2.1. Teen Depression 

Teen depression was measured by the Center for Epidemiologic Studies Depression Scale (CES-D) [34] that asked youth at age 15 how often in the last month did you “feel you could not shake off the blues even with help,” “feel sad,” “feel happy,” “felt life was not worth living,” and “felt depressed.” Response choices were from 0 (*strongly agree*) to 3 (*strongly disagree*) with scores ranging from 0–15. Higher values indicated greater feelings of depression and suicidal ideations [35]. The Cronbach’s alpha coefficient for the current sample was 0.76.

#### 4.2.2. Teen Anxiety

Teen anxiety was measured using the Brief Symptom Inventory 18 (BSI 18) [36]. The six-item scale asked youth at age 15 how often in the last month did you “have spells of terror or panic,” “feel tense,” “feel nervous,” “feel fearful,” “get suddenly scared for no reason,” and “feel restless.” Response choices were from 0 (*strongly agree*) to 3 (*strongly disagree*) with scores ranging from 0–18. Higher values indicated greater feelings of anxiety [35]. The Cronbach’s alpha coefficient for the current sample was 0.76.

#### 4.2.3. Parent Connectedness

Parent connectedness was measured using two items that assessed a parent-teen relationship with respect to closeness and the degree to which a parent and teen talk or share ideas. The two-item scale was adopted from the Family Functioning and the Middle Childhood and Adolescent sections of the National Survey of Children’s Health [37,38]. An adolescent’s closeness to parent was measured by their responses to “How close do you feel to your parent?” Adolescents were also asked “How well do you talk and share ideas with parent?” Responses ranged from 0 (*extremely close/very well*) to 3 (*not very close/not very well*) with scores ranging from 0–6. Higher values represented greater parent connection [35]. The Cronbach’s alpha coefficient for the current sample was 0.74.

#### 4.2.4. School Connectedness

School connectedness was measured using four items that were modified from the PSID-CDS-III [39]. The four-item scale (i.e., “feel a part of school,” “feel close to people at school,” “feel happy to be at school,” and “feel safe at school”) included response choices from 0 (*strongly agree*) to 3 (*strongly disagree*). Scores ranged from 0–12, with higher values representing greater school connection [35]. The Cronbach’s alpha coefficient for the current sample was 0.73.

#### 4.2.5. Neighborhood Connectedness

Neighborhood connectedness was measured using the social cohesion and trust subscale of the neighborhood collective efficacy scale [40]. The four-item subscale asked youth at age 15 if “people around here are willing to help their neighbors,” “this is a close-knit neighborhood,” “people in this neighborhood generally don’t get along with each other,” and “people in this neighborhood do not share the same values.” Response choices were from 0 (*strongly agree*) to 3 (*strongly disagree*) with scores ranging from 0–12. Higher values indicated greater feelings of community connection [35]. The Cronbach’s alpha coefficient for the current sample was 0.68.

#### 4.2.6. Covariates

Gender, age, race/ethnicity, family poverty level, grade level, academic performance, and special education services were control variables used in the study. Gender was a binary variable (0 = male, 1 = female). Age was a continuous variable that was measured in years. Race/ethnicity was a categorical variable that included 0 for White, non-Hispanic, 1 for Black/African American, non-Hispanic, 2 for Hispanic/Latino, 3 for Other, non-Hispanic, and 4 for Multi-racial. Family poverty level was a categorical variable of percentages that represented the ratio of total household income to official poverty thresholds, designated by the U.S. Census Bureau. Response categories included 0 for well below poverty (i.e., 99% or below), 1 for below poverty (i.e., 100–199%), and 2 for above/out of poverty (i.e., 200% or above the federal poverty line). Grade level was a categorical variable that measured the youths’ grade level at Year 15 with 47% of the sample enrolled in the 9th grade. Academic performance was assessed with math grades at the most recent grading period during Year 15. This variable was recoded 0 for low performing (i.e., letter grade of C or lower) and 1 for high performing (i.e., letter grade of A or B). Special education services was a binary variable that was measured from a parent report (0 = no, 1 = yes).

### 4.3. Data Analysis

Missing data patterns were examined and found to be missing at random. List-wise deletion was used to address missingness (<5%). In addition, cases with missing data on key study variables were excluded from analysis [41]. The model assumptions were tested and the results revealed no concerns with issues of multicollinearity; average variance inflation factor (VIF) = 1.26 for teen depression and (VIF) = 1.24 for teen anxiety. Data analytic strategies included the use of descriptive statistics, correlation analysis, and multivariate techniques. 

Hierarchical regression analyses were used to examine the associations of parent connectedness, school connectedness, and neighborhood connectedness with internalizing symptoms. A total of two sets of regression analyses were performed for each dependent variable, depression and anxiety. For each model, in Step 1, depression and anxiety were regressed on statistical controls (i.e., gender, age, race/ethnicity, family poverty level, grade level, academic performance, and special education services). In Step 2, connectedness to social context variables (i.e., family, school, and neighborhood) were added to the models to explain variance in outcomes above and beyond that of the control variables. The coefficient of determination (R^2^) and adjusted R^2^ are reported. Multivariate analyses weighted the sample to be representative of each of the participating cities, adjusting for the oversample of nonmarital births. All of the analyses were conducted using Stata 16.1 software [42].

## 5. Results

### 5.1. Descriptive Information

Table 1 presents the descriptive statistics. Out of the 2590 adolescents that were included in the analysis, 51% were male with an average age of 15.6 years, and 49% Black/African American. Almost 97% of the primary caregivers were youth’s biological mother (90.6%) and biological father (5.7%). Their average age was 41 years, and more than half the sample (58%) fell below 200% of the federal poverty line. Among the teen sample, 72% reported experiencing at least one depressive symptom including suicidal ideations and 86% reported experiencing at least one anxiety symptom within the past month. The average teen depression and anxiety score was 2.93 (*SD* = 2.96) and 4.81 (*SD* = 3.88), respectively. 

### 5.2. Correlations

As shown in Table 2, all of the independent variables were significantly and negatively associated with teen depression and teen anxiety at the *p* < 0.01. All of the significantly associated relationships with teen depression were weak to moderate. The smallest correlation was between neighborhood connectedness and teen depression (*r* = −0.221) and the largest correlation was found between school connectedness and teen depression (*r* = −0.355). For teen anxiety, the largest correlations were found between school connectedness and teen anxiety (*r* = −0.230) and parent connectedness and teen anxiety (*r* = −0.212). Being female from households that were well below the federal poverty line corresponded to increased symptoms of depression and anxiety. 

### 5.3. Hierarchical Regression

Table 3 summarizes the results of the hierarchical regression models predicting teen depression. The hierarchical multiple regression revealed that in Model 1 gender (*β* = 0.168, *p* < 0.001), being Multi-racial (*β* = 0.075, *p* = 0.018), special education services (*β* = 0.162, *p* < 0.001), and poverty thresholds of 100–199% below (*β* = −0.153, *p* = 0.003) and 200% or above the federal poverty line (*β* = −0.149 *p* = 0.006) significantly predicted teen depression, *F*(11, 2578) = 5.47, *p* < 0.001 and accounted for 9% of the variance. Multi-racial, female adolescents were more likely to report depressive symptoms including suicidal ideations compared to White male adolescents. Youth that were receiving special education services in school were more likely to report depressive symptoms compared to youth not receiving special education services. In addition, youth from households that fell well below the federal poverty line (i.e., 99% or below) were more likely to report depressive symptoms compared to adolescents from households that were below the poverty line (i.e., 100–199%) or out of/above the poverty line (i.e., 200% or above).

In Model 2, connectedness to social contextual variables (i.e., family, school, and neighborhood) were added to the model to explain the variance in teen depression beyond that of the control variables. This model explained an additional 11% of the variance in teen depression including suicidal ideation. Parent connectedness (*β* = −0.128, *p* < 0.001), school connectedness (*β* = −0.222, *p* < 0.001), and neighborhood connectedness (*β* = −0.116, *p* = 0.003) significantly predicted teen depression, in addition to the control variables, gender (*β* = 0.128, *p* = 0.004), special education services (*β* = 0.167, *p* = 0.002), and poverty thresholds of 100–199% below the federal poverty line (*β* = −0.169, *p* = 0.002), *R*^2^ = 0.20, *F*(14, 2575) = 12.30, *p* < 0.001. When adding connectedness to social contextual variables to the model, being Multi-racial and from households out of/above the poverty line (i.e., 200% or above) were no longer associated with teen depression. For every unit increase in parent connectedness, teen depression decreased by a score of 0.13, *t*(2575) = −3.35, *p* < 0.001. For every unit increase in school connectedness, teen depression decreased by a score of 0.22, *t*(2575) = −4.59, *p* < 0.001. Also, for every unit increase in neighborhood connectedness, teen depression decreased by a score of 0.12, *t*(2575) = −2.96, *p* = 0.003. 

Table 4 summarizes the results of the hierarchical regression models predicting teen anxiety. The hierarchical multiple regression revealed that in Model 1, special education services (*β* = 0.225, *p* < 0.001) and poverty threshold of 200% or above the federal poverty line (*β* = −0.145, *p* = 0.004) significantly predicted teen anxiety, *F*(11, 2578) = 4.44, *p* < 0.001 and accounted for 8% of the variance. Youth that were receiving special education services in school were more likely to report anxiety symptoms compared to youth that were not receiving special education services. In addition, youth from households that fell well below the federal poverty line (i.e., 99% or below) were more likely to report anxiety symptoms compared to adolescents from households out of/above the poverty line (i.e., 200% or above). 

In Model 2, connectedness to social contextual variables (i.e., family, school, and neighborhood) were added to the model to explain the variance in teen anxiety beyond that of the control variables. This model explained an additional 8% of the variance in teen anxiety. Parent connectedness (*β* = −0.157, *p* < 0.001) and school connectedness (*β* = −0.166, *p* < 0.001) significantly predicted teen anxiety in addition to the control variables, being Black (*β* = −0.118, *p* = 0.043, and special education services (*β* = 0.235, *p* < 0.001), *R*^2^ = 0.16, *F*(14, 2575) = 5.91, *p* < 0.001. For every unit increase in parent connectedness, teen anxiety decreased by a score of 0.16, *t*(2575) = −3.83, *p* < 0.001. For every unit increase in school connectedness, teen anxiety decreased by a score of 0.16, *t*(2575) = −3.22, *p* < 0.001. Also, Black adolescents were more likely to report anxiety symptoms compared to White adolescents.

## 6. Discussion and Implications

This study is distinct in its examination of specific types of connectedness across social contexts in a diverse sample of youth from disadvantaged backgrounds. By examining connectedness to family, school, and neighborhood, this study was able to determine associations between the types of connections and youth depression and anxiety during adolescence. In support of this study’s hypothesis, findings revealed significant associations between youths’ level of connectedness to parent, school, and neighborhood and their depressive symptomology after controlling for gender, age, race/ethnicity, family poverty level, grade level, academic performance, and special education services. More specifically, youth who reported strong connections to their parent, school, and neighborhood were more likely to report lower levels of depressive symptomology, with school connectedness exerting a greater influence. In addition, after holding control variables constant, study findings revealed that youth who reported connections to their parent and school were more likely to report lower levels of anxiety, with each contextual domain exerting relatively the same level of influence. Neighborhood connectedness was not statistically associated with teen anxiety. 

Similar to previous research on the beneficial role of parent-child relationships on youth mental health outcomes [18,25,26], this study provides support for connectedness to parent as an important indicator in the reduction of depressive and anxiety symptomology. Moreover, the findings are meaningful when considering the contextual risk factors that are characteristic of youth born to majority unmarried and low-income parents living in large U.S. cities. Poverty, and the structural factors that perpetuate poverty, tend to have a negative impact on parenting behavior by increasing parenting stress and, in turn, increasing the chances of disengagement or impaired parenting practices [43]. The study findings highlight the resilient nature of youth and their families in the FFCWS dataset whose connectedness appeared to benefit youth despite the at-risk factors. Prior studies have also found that parental support can protect against depression in vulnerable youth [44]. The findings from this study and prior studies support parent-child connectedness as a vital protective factor for disadvantaged youth.

School connectedness was also associated with teen depression and anxiety. These findings replicate previous research that documents the protective impact of schools on adolescent mental well-being [17,31,45]. What is noteworthy is that youth perceptions of school connectedness exerted a greater effect on teen depression above that of family poverty level and other contextual domains. Despite being from low-income backgrounds, connections to schools accounted for more variability in adolescent depressive symptoms. This finding has important practical implications for prevention and intervention efforts and point towards the need for school members to create inclusive and supportive environments that encourage students to form strong connections and to feel safe and cared for with the goal of promoting positive mental well-being, specifically for youth from disadvantaged backgrounds.

A contribution of this study is the examination of neighborhood connectedness on adolescents internalizing symptoms. The findings revealed that youth perceptions of neighborhood connectedness was linked to teen depression, adding to the scant literature on the important role of the neighborhood environment on depressive symptoms during adolescence. However, this finding was not consistent for teen anxiety. The results revealed that neighborhood connectedness was not statistically associated with anxiety symptoms. This finding contradicts previous research that demonstrated empirical support for the relationship between neighborhood connectedness and teen anxiety. For example, [18] found that youth who reported increased connections to their neighborhood were more likely to report reductions in anxiety symptoms. One explanation may be due to differences in studies’ methods, specifically measurements of neighborhood connectedness. Another explanation may be due to the data being largely representative of adolescents from low-socioeconomic households with a higher probability of living in neighborhoods that have a high concentration of poverty and limited resources. As a result, the structural aspects of the neighborhood may be more relevant to adolescent mental well-being than that of perceptions about neighborhood connections. For example, [46] found that neighborhood structural variables (e.g., concentrated poverty, percentage of government assistance, percentage of female-headed families, or unemployment rate) were associated with depression and anxiety symptoms in a sample of adolescents aged 9–15 years. Future research should focus on structural neighborhood processes (e.g., concentrated poverty, number of single-parent households, and racial diversity) in addition to the perceived neighborhood influences when examining adolescent mental health outcomes. 

A key feature of the developmental stage of adolescence is youths’ need to socialize and form connections with their social environments, including family, schools, and communities [21,47]. This distinct feature brings awareness to the significant influence of these daily life encounters on adolescent mental health. The findings from this study highlight the importance of connectedness across social contexts on adolescents internalizing symptoms which have important implications for all youth and not solely youth from disadvantaged backgrounds. First, examining the cumulative impact of social connectedness on adolescents’ depression, including suicidal ideation, allows for a more holistic understanding of its effects on adolescent mental health and well-being which better informs prevention and intervention efforts. Secondly, each social domain exerts an independent influence which may suggest that negative experiences in one environment may be counteracted by more positive ones in others which provides insight on key protective factors. [48] reinforced the protective nature of adolescents’ connections within contextual domains in the prevention of teen depression and suicide by building multiple protective factors to promote positive emotional health. Lastly, in this study youth voices were exemplified by acknowledging how they perceive their daily encounters within their social environment and its direct effect on their mental health. On a broader level, this study provides promising evidence for interventions to be delivered at multiple ecological levels (i.e., family, school, and neighborhood) simultaneously. For example, a cross-system supportive network approach with the school site as a hub bridging parents and community members together and leveraging resources would be optimal in supporting adolescent mental health and well-being. 

## 7. Limitations and Future Directions

This study has several limitations that should be addressed. First, this cross-sectional design limits evidence of causal relationships and the direction of effects. More longitudinal studies are needed to test if connectedness across social domains have a lasting impact on adolescent well-being. Second, data that was used for analysis was self-reported, and increases the risk of reporter bias. Future research should include more objective measures and reporting from different informants such as parents and teachers for triangulation. Third, the measurement of parent connectedness was limited in that it contained only two items and referred to youth connections with their mother. More research that is inclusive of adolescents’ relationships with fathers are vital. In addition, the use of validated measures of parent/family relationship quality would extend this research considerably [49]. This research did not incorporate other important ecological factors such as peer groups. This was a limitation of the data providing no items to accurately assess or reflect peer connectedness. In addition, previous research findings have concluded that there are no significant associations between peer connections and internalizing symptoms [12,18,32]. Future research may need to explore more contextual factors to better account for the variability in teen depression and anxiety symptoms. The findings should be interpreted with caution due to the relatively small size of correlations and regression coefficients.

## 8. Conclusions

Social connectedness during adolescence must remain a key priority with interventions and activities across youths’ social environment that target a reduction of internalizing symptomology. This study demonstrates the importance of parent connectedness, school connectedness, and neighborhood connectedness in the reduction of teen depression including suicidal ideation and the importance of parent connectedness and school connectedness in the reduction of teen anxiety for a diverse sample of adolescents from disadvantaged backgrounds. Some adolescents are at higher risk of mental disorders due to greater exposure and vulnerability to unfavorable social, economic, and environmental circumstances. Taking action to improve the conditions of daily life circumstances and encounters during adolescence is vital and can be enhanced by the social and emotional support and positive interactions with family, schools, and the larger community [8,11].

## Figures and Tables

**Table 1:** Descriptive statistics (*n* = 2590).

Variable	% or M (±SD)
Teen Depression	2.93 (±2.96)
Teen Anxiety	4.81 (±3.88)
Parent Connectedness	4.47 (±1.52)
School Connectedness	9.80 (±2.26)
Neighborhood Connectedness	7.69 (±2.66)
Race	
White [reference]	17%
Black	49%
Hispanic/Latino	26%
Other	3%
Multi-racial	5%
Gender	
Male [reference]	51%
Female	49%
Age	15.60 (±0.76)
Family poverty level	
0–99% [reference]	29%
100–199%	29%
>200%	42%
Grade Level	
6th	<1%
7th	<1%
8th	9%
9th	47%
10th	32%
11th	9%
12th	2%
Academic Performance	
Low [reference]	28%
High	72%
Special Education Services	
No [reference]	88%
Yes	12%

Note: % = percentage for categorical variables; M (SD) = mean (±standard deviation) for continuous variables.

**Table 2:** Correlations among study variables (*n* = 2590).

Variable	1	2	3	4	5
1. Teen Depression	-----				
2. Teen Anxiety	0.647 **	-----			
3. Parent Connectedness	−0.287 **	−0.212 **	-----		
4. School Connectedness	−0.355 **	−0.230 **	0.229 **	-----	
5. Neighborhood Connectedness	−0.221 **	−0.199 **	0.168 **	0.250 **	-----

Note: ** *p* < 0.01.

**Table 3:** Hierarchical regression analysis for teen depression (*n* = 2590).

Model	Variable	*b*	*SE*	*β*	*R* ^2^	*Adjusted R* ^2^	*F*
1					0.091	0.087	5.47 ***
	Gender	0.935	0.233	0.168 ***			
	Age	0.158	0.200	0.034			
	Race (Black)	−0.429	0.302	−0.073			
	Race (Hispanic)	−0.116	0.362	−0.020			
	Race (Other)	−0.201	0.448	−0.015			
	Race (Multi-racial)	0.989	0.418	0.075 *			
	Grade Level	0.051	0.185	0.015			
	Academic Performance	−0.065	0.239	−0.010			
	Special Education Services	1.211	0.369	0.162 ***			
	Below Poverty (100–199%)	−0.984	0.328	−0.153 **			
	Out of/Above Poverty (200% or above)	−0.827	0.300	−0.149 **			
2					0.195	0.190	12.30 ***
	Gender	0.712	0.244	0.128 **			
	Age	0.199	0.197	0.043			
	Race (Black)	−0.623	0.324	−0.106			
	Race (Hispanic)	−0.142	0.361	−0.024			
	Race (Other)	−0.302	0.461	−0.022			
	Race (Multi-racial)	0.774	0.421	0.058			
	Grade Level	0.043	0.206	0.012			
	Academic Performance	0.154	0.260	0.024			
	Special Education Services	1.253	0.404	0.167 **			
	Below Poverty (100–199%)	−1.091	0.351	−0.169 **			
	Out of/Above Poverty (200% or above)	−0.461	0.334	−0.083			
	Parent Connectedness	−0.259	0.077	−0.128 **			
	School Connectedness	−0.292	0.063	−0.222 ***			
	Neighborhood Connectedness	−0.128	0.043	−0.116 **			

Note. For gender, 0 = male, 1 = female; for academic performance, 0 = low, 1 = high; for special education services, 0 = no, 1 = yes. Well below poverty (i.e., 99% or below) was the reference group for family poverty level and White was the reference group for race. * *p* < 0.05; ** *p* < 0.01; *** *p* < 0.001.

**Table 4:** Hierarchical regression analysis for teen anxiety (*n* = 2590).

Model	Variable	*b*	*SE*	*β*	*R* ^2^	*Adjusted R* ^2^	*F*
1					0.082	0.078	4.44 ***
	Gender	0.432	0.342	0.056			
	Age	0.082	0.292	0.013			
	Race (Black)	−0.803	0.468	−0.101			
	Race (Hispanic)	−0.732	0.452	−0.092			
	Race (Other)	−0.820	0.607	−0.045			
	Race (Multi-racial)	0.693	0.914	0.039			
	Grade Level	0.010	0.216	0.002			
	Academic Performance	−0.283	0.348	−0.032			
	Special Education Services	2.29	0.491	0.225 ***			
	Below Poverty (100–199%)	−0.360	0.488	−0.041			
	Out of/Above Poverty (200% or above)	−1.091	0.380	−0.145 **			
2					0.159	0.155	5.91 ***
	Gender	0.167	0.315	0.022			
	Age	0.153	0.274	0.024			
	Race (Black)	−0.939	0.465	−0.118 *			
	Race (Hispanic)	−0.717	0.458	−0.090			
	Race (Other)	−0.948	0.550	−0.052			
	Race (Multi-racial)	0.488	0.834	0.027			
	Grade Level	−0.019	0.225	−0.004			
	Academic Performance	−0.046	0.363	−0.005			
	Special Education Services	2.39	0.496	0.235 ***			
	Below Poverty (100–199%)	−0.501	0.453	−0.057			
	Out of/Above Poverty (200% or above)	−0.670	0.396	−0.089			
	Parent Connectedness	−0.434	0.113	−0.157 ***			
	School Connectedness	−0.297	0.089	−0.166 ***			
	Neighborhood Connectedness	−0.123	0.072	−0.082			

Note. For gender, 0 = male, 1 = female; for academic performance, 0 = low, 1 = high; for special education services, 0 = no, 1 = yes. Well below poverty (i.e., 99% or below) was the reference group for family poverty level and White was the reference group for race. * *p* < 0.05; ** *p* < 0.01; *** *p* < 0.001.

## Data Availability

This research was based on public data from the Fragile Families and Child Wellbeing Study (FFCWS) that was provided by Princeton University’s Center for Research on Child Wellbeing (CRCW) and the Columbia Population Research Center (CPRC). These data can be found here: https://fragilefamilies.princeton.edu/documentation (accessed on: 15 August 2021). The views and opinions expressed in this paper are those of the author and do not represent the views of CRCW and CPRC.
